# The sex-specific transcriptome of the hermaphrodite sparid sharpsnout seabream (*Diplodus puntazzo*)

**DOI:** 10.1186/1471-2164-15-655

**Published:** 2014-08-06

**Authors:** Tereza Manousaki, Alexandros Tsakogiannis, Jacques Lagnel, Elena Sarropoulou, Jenny Z Xiang, Nikos Papandroulakis, Constantinos C Mylonas, Costas S Tsigenopoulos

**Affiliations:** Institute of Marine Biology, Biotechnology and Aquaculture (I.M.B.B.C.), Hellenic Centre for Marine Research (H.C.M.R.), Heraklion, Greece; Genomics Resources Core Facility, Weill Cornell Medical College, New York, USA

**Keywords:** Sparidae, Sharpsnout seabream, *Diplodus puntazzo*, RNA-Seq, Transcriptome, Gonads, Brain, Sex differentiation, Hermaphroditism

## Abstract

**Background:**

Teleosts are characterized by a remarkable breadth of sexual mechanisms including various forms of hermaphroditism. Sparidae is a fish family exhibiting gonochorism or hermaphroditism even in closely related species. The sparid *Diplodus puntazzo* (sharpsnout seabream), exhibits rudimentary hermaphroditism characterized by intersexual immature gonads but single-sex mature ones. Apart from the intriguing reproductive biology, it is economically important with a continuously growing aquaculture in the Mediterranean Sea, but limited available genetic resources. Our aim was to characterize the expressed transcriptome of gonads and brains through RNA-Sequencing and explore the properties of genes that exhibit sex-biased expression profiles.

**Results:**

Through RNA-Sequencing we obtained an assembled transcriptome of 82,331 loci. The expression analysis uncovered remarkable differences between male and female gonads, while male and female brains were almost identical. Focused search for known targets of sex determination and differentiation in vertebrates built the sex-specific expression profile of sharpsnout seabream. Finally, a thorough genetic marker discovery pipeline led to the retrieval of 85,189 SNPs and 29,076 microsatellites enriching the available genetic markers for this species.

**Conclusions:**

We obtained a nearly complete source of transcriptomic sequence as well as marker information for sharpsnout seabream, laying the ground for understanding the complex process of sex differentiation of this economically valuable species. The genes involved include known candidates from other vertebrate species, suggesting a conservation of the toolkit between gonochorists and hermaphrodites.

**Electronic supplementary material:**

The online version of this article (doi:10.1186/1471-2164-15-655) contains supplementary material, which is available to authorized users.

## Background

Teleosts exhibit remarkably diverse patterns of sex modes. The way males and females develop, and the molecular mechanisms underlying those differences, vary dramatically among taxa. In teleosts, the decision on an individual’s sex (‘sex determination’) may be due to genetic and/or environmental factors [1–3] with an evident epigenetic component [4]. Apart from the genes that determine sex, downstream genes and pathways drive the development and maintenance of sex-specific phenotypes. Those processes define sex differentiation. The genes involved in sex determination and differentiation form the necessary toolkit leading to the sex-specific phenotypes. However, the picture becomes more complicated in cases of hermaphroditism, a rather common sexual system among teleosts.

The molecular processes underlying sex have been deeply studied in model vertebrates like human, mouse, chicken and African clawed frog [5–8]. Several studies on fish have unveiled the genes responsible for sex determination in gonochoristic species, such as *Dmy* in medaka [9, 10], *Amhr2* in Tiger pufferfish [11], *Sdy* in Rainbow trout [12] and *Amhy* in Patagonian pejerrey [13]. Other studies have revealed loci linked to sex in various species (e.g. [14] for stickleback; [15] for tilapia; [16] for Atlantic halibut; [17] for turbot; [18] for zebrafish), even in the hermaphrodite Gilthead seabream [19, 20]. All these studies illustrate the large diversity in sex determination among teleosts. However, the genes involved in sex differentiation are considered conserved across vertebrates [21, 22], even though alternative scenarios have also been suggested [23]. To get an overview of the genetic toolkit deployed for the development and maintenance of the differences between sexes, whole-transcriptome approaches are required [24]. Several transcriptomic analyses have characterized the expression differences that underlie the two sex phenotypes in fish (e.g. [25–30]) and revealed that the main pathways are present in most species, although the role of each gene might change.

Most studies on understanding sex differentiation have been conducted on gonochoristic taxa. Hermaphroditism, however, is common among teleosts and has evolved repeatedly in different lineages [31]. The two sexual systems are sometimes observed even among phylogenetically-close species, suggesting that the molecular pathways involved might not differ dramatically. Sparidae is a teleost family with a wide variety of sex mechanisms [32–34]. Sparids exhibit either gonochorism or various forms of hermaphroditism, such as simultaneous, sequential or rudimentary hermaphroditism (simultaneous: presence of both male and female gonads; sequential: an individual develops first as a functional male and then changes to female or vice versa; rudimentary: immature individuals carry both male and female immature gonad types and during maturation one of the two types develops fully, determining the sex). A recent study on the ancestral reconstruction of sexual patterns in sparids revealed both gonochorism and hermaphroditism in almost every group of the family [31]. Therefore, there is a great potential for understanding the molecular mechanisms underlying gonochorism and hermaphroditism within Sparidae [31, 35]. To date, considerable effort to unravel the genes involved in sex determination, sex differentiation and sex change have been conducted mainly on the protandrous black porgy, *Acanthopagrus schlegelii* [35–42]. Further knowledge comes from QTL studies on the protandrous Gilthead seabream, *Sparus aurata* [19, 20], but current knowledge concerning other Sparidae species is poor.

The Sharpsnout seabream, *Diplodus puntazzo*, is a sparid of great importance for the industry of fisheries and aquaculture. This species is also particularly interesting from an evolutionary point of view, as it has one of the most spectacular reproductive systems; it is rudimentary hermaphrodite with some instances of protandry [43, 44]. Several studies have investigated various aspects of its biology, including reproduction and development (e.g. [44–46]), but the available information concerning the species’ genetic content is limited. To date, this is the case for most sparids, except for the two protandrous species of *Sparus aurata* and *Acanthopagrus schlegelii*, which account for more than 90% of the Sparidae sequences available in GenBank. With modern sequencing technologies, this lack of knowledge can be overcome and ultimately allow the comparison of the genetic networks involved in sex determination and differentiation among closely related species that exhibit different reproductive modes.

Here, we sought to identify and understand the molecular toolkit underlying the sex differences of the expressed transcriptome in the rudimentary hermaphrodite sharpsnout seabream. To that end, we employed an RNA-Sequencing (RNA-Seq) approach [47] aiming at capturing the gene content of sharpsnout seabream that exhibits sex-biased expression pattern. We chose to study both gonad and brain tissues. Gonads were selected to get a comprehensive overview of the genes responsible for the divergence of the primary sex-related structure; brain was included to increase the species genic information and to understand how sex affects brain functions, as shown in gonochoristic fishes (see [48] and references therein). Our results revealed major expression differences between male and female gonads, but only minor differences between male and female brains. Most genes involved in primary pathways of sex determination and differentiation in other vertebrates are present also in the sharpsnout seabream; this implies a conservation of pathways between gonochorists and hermaphrodites. Finally, we constructed a dataset of genes and a resource of genetic markers that will assist future genetic research at the species and family level.

## Methods

### Sample collection

Animal care was carried out according to the “Guidelines for the treatment of animals in behavioural research and teaching” [49]. Hatchery produced sharpsnout seabream (from eggs spawned in October 2010) were reared in tanks supplied with flow-through seawater under ambient conditions at the Institute of Marine Biology, Biotechnology and Aquaculture. Fish were fed daily to apparent satiation using a commercial extruded feed (SKRETTING, Norway and IRIDA S.A., Greece). During the reproductive period (October-December) of 2012 (when fish were 2+ years old), fish were sampled randomly from the population and were euthanized in a mixture of seawater and ice. Selected individuals were examined for sexual maturation, based on the presence of releasable sperm from the males or the presence of vitellogenic oocytes in the females. At that time, sperm could not be collected from any of the males, but histological evaluation indicated the presence of intratesticular spermatozoa. Females were immature, containing only primary oocytes in their ovaries.

Gonad and brain tissues were sampled from four male and four female individuals (16 samples in total) in a sterile and RNase-free way and fixed immediately in RNAlater (Applied Biosystems, Foster City, CA, USA). Tissues for RNA extraction were stored at 4°C overnight and then transferred to -80°C until further processing.

### RNA extraction and sequencing

To avoid biases regarding different expression in different parts of the same organ, we homogenized the whole brain and male gonad samples whereas in the case of female gonads, because of their large size (1.2 g - 4.3 g) we excised three different parts of the organ (the posterior, the anterior and the middle tissue) and homogenized it in QIAzol Lysis buffer (QIAGEN^®^) following supplier’s recommendations.

The disruption and homogenization occurred using TissueLyser II (QIAGEN^®^) and steal beads of 5 mm diameter (QIAGEN^®^). We used Qiagen’s miRNeasy extraction kit, as we targeted on extracting both mRNA and miRNA (only mRNA was analyzed in this study). The quantity of the isolated RNA was measured spectrophotometrically with NanoDrop^®^ ND-1000 (Thermo Scientific), while its quality was tested on an agarose gel (electrophoresis in 1.5% w/v) and further on an Agilent Technologies 2100 Bioanalyzer (Agilent Technologies). The majority of the samples had an RNA Integrity Number (RIN) value higher than 8 (Table 1). This was not the case for any of the female gonad samples. Further effort to improve RNA extraction did not yield any higher RIN value. Although NanoDrop values were of the expected range (260/280 ratio [1.98-2.07]), the agarose gel and Bioanalyzer revealed the presence of an enormous peak at 100 bp (Additional file 1: Figure S1). However, several teleost female gonads show a similar RNA profile as reported earlier [50]. This high peak corresponds to 5S rRNA and possibly hampers the correct estimation of the 18S and 28S rRNA peaks. This was consistent in all female gonad extractions and possibly affected the RIN number as the observed pattern deviated greatly from the expected pattern of RNA content.

**Table 1:** **Sequencing summary**

Females id	tissue	RIN	Raw reads	Filtered paired reads	
				Total	Mapped
1	gonad	N/A	20961364	11297594	9831370
1	brain	9.3	23980450	13193988	11095910
2	gonad	N/A	27194286	14697094	12745828
2	brain	8.7	25146494	13764286	11511286
3	gonad	2.5	27629298	14487792	12602744
3	brain	8.9	31171108	16631824	13782126
4	gonad	2.5	36169194	18753838	16134938
4	brain	N/A	27213654	14519532	12227478
**Males id**	**tissue**	**RIN**	**Raw reads**	**Total**	**Mapped**
5	gonad	9.4	24782894	12751168	10650100
5	brain	9.2	23058518	12279090	10263134
6	gonad	N/A	28182238	15353332	12958232
6	brain	9	28819914	15465380	13044458
7	gonad	8.8	28895952	15281070	12634914
7	brain	8.4	17381152	9535140	8262496
8	gonad	9.5	28583256	15336768	12479306
8	brain	9.4	30818278	16496042	13791216
Total	-	-	429988050	229843938	194015536

Finally, all 16 samples were used for library preparation and sequencing as 100 bp paired reads in 1.5 lanes of a HiSeq2500 following the protocols of Illumina Inc. (San Diego, CA) in the Genomics Resources Core Facility of Weill Cornell Medical College.

### Read pre-processing

Read quality was assessed with FastQC [51] and subjected to quality control with FASTX_Toolkit [52]. Adapters were trimmed with fastx_clipper. Quality trimming was conducted with fastq_quality_trimmer (minimum quality 25, minimum read length 50). Reads were further quality controlled by excluding those with more than 5% of low-quality (quality threshold 25) nucleotides using fastq_quality_filter. After filtering, read pairs were reconstructed with a custom Perl script.

### Transcriptome assembly and annotation

For the assembly, we pooled the filtered reads of all samples and implemented three different trials using SOAP-DENOVO [53] (kmer = 35, min length 200 nucleotides), Velvet/Oases [54, 55]) (kmer = 35, min length 200 nucleotides) and Trinity [56] (trinityrnaseq_r2013-02-25; default kmer 25, min length 200 nucleotides). The three candidate assemblies were evaluated by BLASTn [57] against *Oreochromis niloticus*, *Oryzias latipes* and *Gasterosteus aculeatus* cDNA dataset downloaded from Ensembl database [58] with an *e*-value threshold of 10^-9^. The assembly produced by Trinity had the highest number of significant similarity with unique genes from all three teleost species and was selected.

To assess the assembled transcripts and exclude the spurious ones, we pooled all the reads and mapped them to the selected assembly with Bowtie [59] within RSEM [60] using the script available in trinity utilities *run_RSEM_align_n_estimate.pl*. Putative transcripts with less than 1% of a locus reads mapped to that particular isoform (IsoPct < 1) were eliminated (as proposed in [61]). The same was done for those with Fragments Per Kilobase of transcript per Million mapped reads (FPKM) values less than 0.3. The choice of FPKM threshold was based on BLASTn similarity searches (*e*-value threshold 10^-10^) against *O. niloticus* cDNA sequences. The unfiltered transcripts dataset had significant similarity with 18,527 *O. niloticus* genes, while the filtered assembly had significant similarity with 17,346 out of 21,462 genes reported in Ensembl. This dataset constituted the final assembled transcriptome given that the selected threshold value resulted in elimination of 255,891 possibly spurious transcripts, while the hits to unique genes of *O. niloticus* were only slightly reduced.

To annotate the assembled transcripts, we conducted a BLASTx similarity search against the NCBI protein database *nr* (*e*-value threshold 10^-9^; keeping the top ten hits). BLASTx was done in parallel using NOBlast [62]. The output was used in Blast2GO [63] where gene ontology terms were retrieved and assigned to the transcripts (only the longest transcript was used per locus). The open reading frames (ORF) were extracted per sequence with the EMBOSS program *getorf*. InterProScan [64] was run on the longest ORF per transcript. The run was done in parallel splitting the query in 100 subqueries and merging the output with custom scripts. GO terms derived from InterProScan were merged with GO terms derived from BLASTx against *nr*. In cases where accurate orthology inference was of interest (e.g. for identifying the orthologs of the genes associated with sex in other taxa), we implemented a reciprocal BLASTn hit approach of the annotated sequences of medaka, tilapia and other fish from Ensembl against sharpsnout seabream assembled transcripts. When the gene of interest had significant similarity with the assembled transcripts, we used the top hit in a BLASTn search towards the transcriptome of the starting species. If that step returned the initial transcript, we assumed orthology between the two.

### Differential expression

The paired reads of each sample were mapped to the assembly with Bowtie and abundance was estimated with RSEM v. 1.2.4, as implemented in the trinity script *run_RSEM_align_n_estimate.pl*. The estimated expected counts for each sample, at the gene level, were extracted and used for the analysis of differential expression conducted in DESeq [65], a software considered accurate and conservative for differential expression analyses [66, 67]. Samples were grouped according to sex and expression was compared for each tissue separately, following the developers’ manual (FDR threshold of 0.05). Due to a reported difficulty in assessing differential expression when a gene is expressed only in one group [66], we considered as significant those genes only when the expression in the other group was higher than 30 normalized counts in total.

### Detection of genetic markers

The assembled sequences were scanned for microsatellites with Phobos [68]. We detected non-exact Short Tandem Repeats (STRs) with 2–10 repeat unit length and a minimum length of 20 nucleotides. A custom Perl script was used to parse the output.

Single Nucleotide Polymorphisms (SNPs) were detected with SAMTools [69] and VCFtools [70]. Alignment (.bam) files produced through mapping for gonads and brain were merged for each individual. Alignment files were further filtered for mapping quality (threshold 75), number of mismatches to the reference (‘ΝΜ’ threshold 10) and for proper pair mapping with BamTools [71]. Then, they were sorted and piled (with SAMTools function ‘mpileup’) to detect candidate SNPs. Custom Perl scripts were used to eliminate SNPs that formed clusters (>1 variant every 4 bases) to exclude hypervariable regions where accurate alignment is difficult. Those with SNP QUAL < 25, total high quality bases coverage below 15 and minor allele read ratio < 0.2 were excluded [72]. In each locus we kept only the SNPs belonging to the transcript with the highest SNP number. Finally, SNPs were categorized to synonymous, non-synonymous and those that belong to the transcripts UTRs. For that, we first retrieved the predicted ORF per transcript with the highest similarity to tilapia proteins (*e*-value threshold 10^-10^). Then, we evaluated whether each SNP is located within the coding region defined by the ORF, and for those located within the coding region if it causes a synonymous or a non-synonymous mutation using a Perl script.

To identify SNPs for which genotyping can be conducted robustly we applied extra filtering steps. Those included the genotype quality (‘GQ’) score > =20 as provided by SAMtools and VCFtools pipeline and required minimum coverage of the SNP site per sample > 5 for all samples. Note that the genotype quality is a function of the probability that the genotype call is wrong given that the site is variable. SNPs that passed the extra criteria were used for a PCA and a relatedness analysis with the R package SNPRelate [73]. For the Relatedness analysis, we estimated Identity By Descent with the Maximum Likelihood method and calculated the kinship coefficient. Finally, all scripts used in the study are available upon request.

## Results

### Sharpsnout seabream assembled transcriptome

Illumina HiSeq2500 sequencing yielded 429,988,050 paired reads (214,994,025 read pairs) (Table 1). The filtering process resulted in 229,843,938 paired and 74,027,237 single or orphan high quality reads used for the transcriptome assembly.

**Figure 1:**
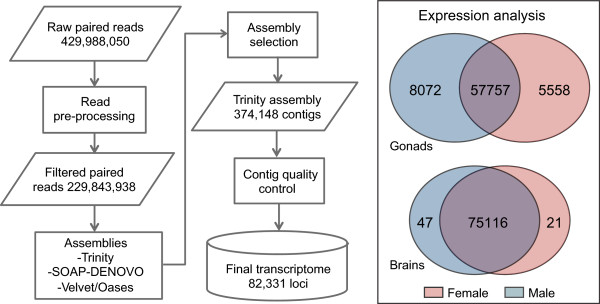
**The pipeline followed to build the assembly and the male** ***versus*** **female expression profiles.** On the left, flow chart of the steps implemented from raw reads to the selection of the final assembled loci. On the right, Venn diagram showing the loci commonly and differentially expressed in the two sexes for brains and gonads.

The initial assembly process produced 374,149 putative transcripts (N50: 1,712 nucleotides, mean length: 871 nucleotides). Following further editing and transcript quality assessment (see Methods), we limited our dataset to 118,258 transcripts belonging to 82,331 loci (N50: 2,092; mean length: 1,352 nucleotides) (Figure 1). The unfiltered transcripts dataset had significant similarity to 18,527 *O. niloticus* genes, while the filtered assembly had significant similarity to 17,346 out of 21,462 genes reported in Ensembl. Given that the filtering resulted in elimination of 255,891 possibly spurious transcripts, while the hits to unique genes of *O. niloticus* were only slightly reduced, the restricted dataset constituted the final transcriptome. Out of the 82,331 loci, 31,895 had significant BLASTx similarity hits with publicly available protein sequences, 22,574 of the loci were assigned GO terms and 40,823 had an InterProScan protein domain match. The great majority of the top-hits of the assembled transcripts had significant similarity with sequences of *Maylandia zebra*, *Oreochromis niloticus*, *Takifugu rubripes*, *Oryzias latipes* and *Dicentrarchus labrax*. Further, 14,041 of the genes without any significant similarity to known proteins had InterProScan protein domains assigned.

### Comparative expression profiling between males and females

**Figure 2:**
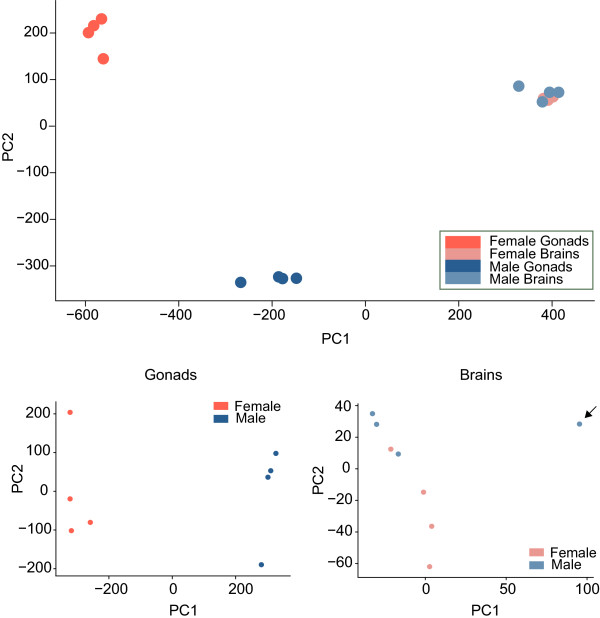
**Principal component analysis of the expression profiles.** On top, the global expression profiles of gonad and brain samples of male and female individuals analyzed in a PCA. At the bottom, brain and gonad samples are analyzed separately. The brain sample pointed by the arrow is considered an outlier and was excluded from the differential expression analyses.

The global gene expression pattern observed in the tissues of the eight individuals used in this study is summarized in a principal component analysis (PCA) (Figure 2). The picture obtained shows a clear separation between female and male gonads with remarkable variation within the two groups. On the contrary, brain expression patterns are similar between males and females in this general overview.

A separate PCA of the brain global expression values showed a scattered pattern with some overlap between the groups of males and females (Figure 2). Notably, one of the male brain samples had an extreme profile compared to the other male or female samples. To test whether this was due to library construction error, we mapped the reads to *Sparus aurata* ribosomal RNA (*r*RNA) to find no relation of *r*RNA content to the outlier (for the majority of samples 0–0.05% of the reads were mapped on *r*RNA). Thus, this sample was excluded from the differential expression analysis. For gonads, global expression patterns showed once again a clear separation of males and females (Figure 2). Sex-specific expression patterns were evaluated separately for brain and gonad tissues and are described below.

#### Male vs. female gonads expression patterns

**Figure 3:**
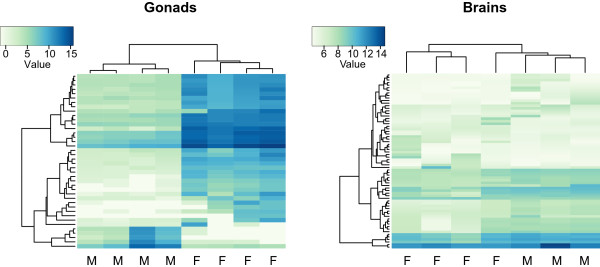
**Heatmap of the differentially expressed genes for gonad and brain samples.** Heatmap of the variance-stabilized transformed count data of gonad and brain samples for the differentially expressed genes between male and female individuals. Note that only the top 40 genes are included in the gonad heatmap; for the brain heatmap all genes are included.

In gonads, male and female expression patterns differed greatly. Out of the total 82,331 loci, 71,387 had an estimated abundance of at least one pair of reads in gonad samples. The differential expression analysis revealed 5,558 loci up-regulated in female and 8,072 loci up-regulated in male gonads (Figure 1; Additional file 2: Figure S2; Additional file 3: Table S1). Clustering of the samples based on the top 40 differentially expressed genes grouped males and females separately (Figure 3).

**Figure 4:**
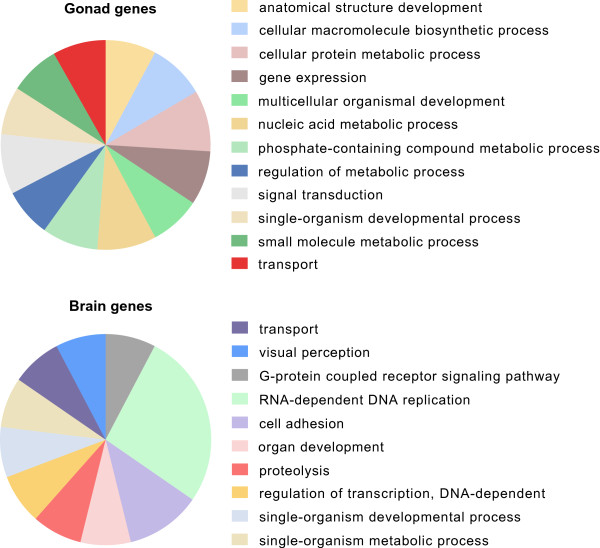
**Gene Ontology profiling of gonad and brain samples.** Pie charts representing the GO terms (Biological Process) associated with the genes over-expressed in gonad and brain samples. For gonads only terms with > 700 sequences are shown.

The putative functions of those genes include development, signal transduction and metabolism (Figure 4). To test whether certain functions are more frequently observed in one of the two sexes, we conducted a Fisher’s exact test in Blast2GO comparing the GO terms of the male versus the female over-expressed genes (each gene was represented by the longest transcript; *p*-value 0.05; Table 2; see Additional file 4: Table S2 for complete list). The main GO terms associated with the genes over-expressed in females are involved in metabolism, while in males in signaling and regulation of transcription.

**Table 2:** **Comparison of male** ***versus*** **female up-regulated gene functions**

GO-ID	GO-Term	FDR	P-Value	Females seq. count	Males seq. count	Over-represented
GO:0006412	translation	7.8537E-48	9.13693E-51	186	6	females
GO:0008152	metabolic process	3.14977E-46	4.07157E-49	1707	1184	females
GO:0022613	ribonucleoprotein complex biogenesis	3.00773E-35	7.77593E-38	123	1	females
GO:0071704	organic substance metabolic process	2.96565E-34	8.05049E-37	1497	1058	females
GO:0044237	cellular metabolic process	5.57895E-34	1.58657E-36	1392	959	females
GO:0010467	gene expression	7.71755E-34	2.29451E-36	549	240	females
GO:0042254	ribosome biogenesis	1.02323E-31	3.3067E-34	106	0	females
GO:0044238	primary metabolic process	1.6642E-31	5.80837E-34	1445	1028	females
GO:0006396	RNA processing	3.75931E-31	1.40926E-33	146	11	females
GO:0043170	macromolecule metabolic process	1.84976E-26	9.08619E-29	1119	758	females
GO:0007154	cell communication	2.14665E-29	8.87965E-32	289	641	males
GO:0044700	single organism signaling	2.48667E-29	1.0929E-31	280	628	males
GO:0023052	signaling	2.48667E-29	1.0929E-31	280	628	males
GO:0007165	signal transduction	1.98929E-28	9.00013E-31	269	606	males

Fisher’s exact test showing top GO terms significantly over-represented in loci up-regulated in male or female gonad samples. For a complete list see Additional file 4: Table S2.

#### Sex determination and differentiation genes in gonads

The expression profiles of genes known to be involved in sex determination and differentiation (as reviewed in [28]) were closely examined (Table 3). Orthology with other genes was assessed with the reciprocal best hit approach (See Methods). Out of 44 target genes, 42 are found within the assembled transcriptome. The only genes not found were *Fgf9* and *Dmrt6. Dmr6* is absent from the teleost lineage according to Ensembl orthology inference and *Fgf9* might be not expressed, not sequenced or lost from sharpsnout seabream. Sixteen genes are significantly differentially expressed between the two gonad types, while 15 change in the ‘expected’ direction of expression, assuming that the expression direction observed in other vertebrates is conserved in sharpsnout seabream. Only *Dhh* shows an overexpression in female gonads, opposite to the expected.

**Table 3:** **Sharpsnout seabream genes involved in sex determination and differentiation in other vertebrate taxa**

Starting sequence	Species	Gene name	Gene description	Locus	Exp. up-regulation	Obs. up-regulation
ENSORLT00000015555	medaka	*Wt1*	Wilm’s tumour suppressor-1	comp117315_c1	M/F	M
ENSORLT00000012371	medaka	*Wt1b*	Wilm’s tumour suppressor-1	comp117315_c1	M/F	M
ENSORLT00000014817	medaka	*Dax1*	dosage-sensitive sex-reversal-adrenal hypoplasia congenital-critical region of X chromosome, gene 1	comp87968_c1	M	-
ENSXMAT00000015647	platyfish	*Dmrt1*	double sex and mab-3 related transcription factor 1	comp110196_c1	M	M
ENSONIT00000017862	tilapia	*Dmrt3*	double sex and mab-3 related transcription factor 3	comp88038_c1	M	M
ENSLOCT00000007854	spotted gar	*Dmrt6/Dmrtb1*	double sex and mab-3 related transcription factor 6	-	M	-
ENSORLT00000021733	medaka	*Gata-4*	GATA-binding protein 4	comp114090_c1	M/F	M
ENSORLT00000025798	medaka	*Fgf9*	fibroblast growth factor 9	-	M	-
ENSORLT00000012578	medaka	*Fgf20*	fibroblast growth factor 20	comp85925_c1	M	-
ENSONIT00000015290	tilapia	*Dhh*	desert hedgehog	comp116302_c0	M	F
ENSONIT00000006022	tilapia	*Amh*	anti-Mullerian hormone	comp107049_c0	M	M
ENSONIT00000015711	tilapia	*Amhr2*	anti-Mullerian hormone receptor, type II	comp111277_c1	M	-
ENSONIT00000022132	tilapia	*Ar*	androgen receptor	comp108409_c2	M	M
ENSONIT00000016191	tilapia	*Arb*	androgen receptor beta	comp122243_c0	M	-
ENSONIT00000025518	tilapia	*Wnt4*	Wingless-type MMTV integration site family 4	comp94275_c1	F	-
ENSONIT00000016320	tilapia	*Wnt4b*	Wingless-type MMTV integration site family 4 b	comp308556_c0	F	-
ENSONIT00000020541	tilapia	*Rspo-1*	R-spondin-1	comp102190_c3	F	-
ENSONIT00000009116	tilapia	*Ctnnb1*	catenin β-1	comp112822_c5	F	F
ENSONIT00000026114	tilapia	*Foxl2*	forkhead box transcription factor L2	comp95964_c0	F	-
ENSONIT00000017958	tilapia	*Fst*	follistatin	comp112642_c0	F	-
ENSONIT00000000198	tilapia	*Cyp19a1*	aromatase α	comp117077_c0	F	F
ENSONIT00000008152	tilapia	*Cyp11b*	11beta-hydroxylase	comp108630_c0	M	M
ENSORLT00000018193	medaka	*Esr1*	oestrogen receptor 1	comp117911_c10	F	-
ENSORLT00000013323	medaka	*Erα*	oestrogen receptor α	comp112646_c1	F	-
ENSORLT00000022182	medaka	*Erβ*	oestrogen receptor β	comp112065_c1	F	-
ENSORLT00000022555	medaka	*Erβ2*	oestrogen receptor β 2	comp108758_c0	F	-
ENSORLT00000009986	medaka	*Sox9b*	SRY-related HMG-box 9	comp113806_c6	M	-
ENSORLT00000023249	medaka	*Sox9*	SRY-related HMG-box 9	comp108517_c9	M	-
ENSONIT00000006616	tilapia	*Sox8*	SRY-related HMG-box 8	comp113806_c6	M	-
ENSONIT00000024737	tilapia	*Sox8a*	SRY-related HMG-box 8	comp70271_c1	M	-
ENSONIT00000010558	tilapia	*Sox10*	SRY-related HMG-box 10	comp116475_c6	M	-
ENSONIT00000022743	tilapia	*Sox10(2 of 2)*	SRY-related HMG-box 10	comp114808_c13	M	-
ENSONIT00000009624	tilapia	*Gsdf*	Gonadal soma derived factor	comp113923_c0	M	-
ENSONIT00000004791	tilapia	*Pdgfaa*	platelet-derived growth factor alpha a	comp113241_c2	M	M
ENSONIT00000006486	tilapia	*Pdgfab*	platelet-derived growth factor alpha b	comp107178_c1	M	-
ENSONIT00000016484	tilapia	*Pdgfba*	platelet-derived growth factor beta a	comp117159_c4	M	-
ENSONIT00000010783	tilapia	*Pdgfbb*	platelet-derived growth factor beta b	comp113875_c0	M	M
ENSONIT00000016453	tilapia	*Pdgfrb2*	platelet-derived growth factor receptor, beta	comp104719_c1	M	M
ENSONIT00000022434	tilapia	*Pdgfrb1*	platelet-derived growth factor receptor, beta	comp113376_c0	M	-
ENSONIT00000003760	tilapia	*Pdgfra*	platelet-derived growth factor receptor, alpha	comp116299_c0	M	M
ENSONIT00000025493	tilapia	*Sf1*	steroidogenic factor-1	comp120045_c3	M/F	M
ENSONIT00000009791	tilapia	*Srd5a1*	steroid-5-alpha-reductase, alpha polypeptide 1	comp121181_c8	M	-
ENSONIT00000008643	tilapia	*Srd5a2*	5α-reductase 2	comp106131_c0	M	-
ENSORLT00000000749	medaka	*Srd5a3*	5α-reductase 3	comp115080_c0	M	-

The expected direction of expression (Exp. Up-regulation) is estimated from previous reports on other vertebrates, while the observed (Obs. Up-regulation) is the direction found in sharpsnout seabream gonads. M: over-expression observed in males, F: over-expression observed in females.

Further data mining revealed eight loci containing the GO term “sex differentiation” or “sex determination”, and 34 more related to the terms “female” or “male” (e.g. female/male gonad development, female/male meiosis, female pregnancy, etc.). From those 42 genes in total, 17 were significantly differentially expressed in the gonads (Additional file 5: Table S3) including *Dmrt1* and *Ctnnb1*.

To search for potential genes that are present or absent from the two gonad tissues, we listed those differentially expressed loci that had an estimated abundance of absolutely zero counts in one gonad type, but at least a minimum of expression in the other (mean count > 30). We found 95 loci fulfilling those criteria (Additional file 6: Table S4). The majority (89 out of 95) exhibited a male-biased expression. Those loci were used in a similarity search (BLASTp) against tilapia proteins (*e*-value threshold 10^-10^). Our search retrieved 21 tilapia genes as top hits (Additional file 6: Table S4). Through Ensembl BioMART interface, we retrieved the scaffolds those genes are located in tilapia and downloaded the orthologous genes for stickleback and human. This comparison showed that some of the tilapia genes are located in the same scaffold. Search in stickleback showed that some of the genes located in different scaffolds in tilapia are found in the same chromosome in stickleback. Finally, the human ortholog of the genes *Fam70A* and *Xpnpep2* are both located on the X chromosome (Additional file 6: Table S4).

#### Male vs. female brain expression patterns

In brain, 75,184 loci had an estimated abundance of more than one pair of reads. Male and female overall expression patterns were indistinguishable (Figure 2). However, the comparison of brain expression patterns (after excluding the outlier sample) between males and females revealed 68 genes (Figure 1; Additional file 3: Table S1) with significant expression difference between the two sexes (21 were over-expressed in females and 47 in males). Clustering of the samples based on all differentially expressed genes led to the grouping of one female with the males as observed also in the PCA conducted in total expression profiles (Figure 3; Figure 2).The main GO terms associated with the genes found in brain comparison are related to transposable elements, developmental processes, visual perception and signaling among others (Figure 4). Fisher’s exact test did not reveal any significantly over-represented term in the brain dataset compared to the whole assembly, probably due to the small size of the dataset.

### Genetic marker discovery

**Figure 5:**
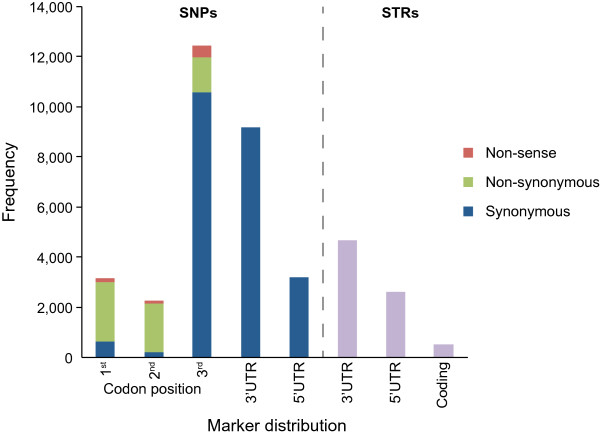
**Genetic markers distribution at the coding loci.** SNPs and STRs within coding regions are categorized according to their distribution at the codon position (for SNPs only) or 5′ and 3′UTRs.

We searched for two types of markers, SNPs and STRs. For the SNP discovery, we combined the reads obtained from the brain and gonad of each individual to increase the individual coverage and then applied various quality filters (see Methods, Table 4). In total, we found 85,189 SNPs, that pass the quality criteria, located in 30,291 loci (Additional file 7: Table S5). From those loci, 9,997 are significantly similar to known tilapia cDNA sequences and contain at least one predicted ORF. From the total SNPs within those 9,997 loci (30,278 SNPs), ~10% were in the first, ~7% in the second and ~41% in the third codon position. The rest were in the non-coding regions (5′ and 3′ UTRs) (Figure 5).

**Table 4:** **SNP discovery filters**

Filtering summary		
Criteria	SNPs	Loci
Overall	354323	79898
Delete clusters	308353	78721
Quality > 25	244875	53911
Coverage > 15	149232	36829
Read allele ratio > 0.2	96942	30291
1 transcript per locus	85189	30291

In each step the remaining SNPs and the corresponding loci are shown.

From the loci containing SNPs, ~25% were significantly differentiated in males and females (37 in brain and 7,592 in gonads). Especially for the gonads, in which the total number of differentially expressed genes was 13,630, this is way more than expected by chance (two-sample *z* test on proportions; *p*-value < 0.05).

The required coverage and quality criteria applied secure a robust genetic marker discovery. In an attempt to genotype all individuals in certain loci, we applied further criteria requiring certain depth and quality per individual SNP calling (see Methods). Those filters reduced dramatically the number of SNPs that are exploitable for genotyping to 1,009 SNPs (Additional file 8: Table S6). The individual genotypes for all these SNPs were used in a PCA (Additional file 9: Figure S3) and a relatedness analysis (Additional file 10: Table S7). The average kinship coefficient (probability that two alleles randomly chosen from two individuals are Identical By Descent) between pairs is ~0.10 (min: 0.04; max: 0.18). No parent-offspring relationship is observed (expected kinship coefficient 0.25). Note that linkage disequilibrium-based SNP pruning was not conducted due to lack of information regarding linkage of SNPs.

**Figure 6:**
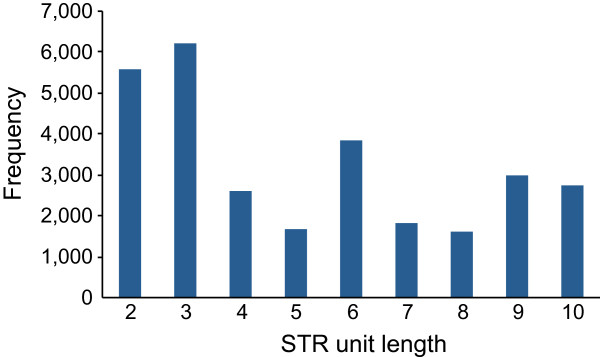
**Unit length distribution of identified STRs.** Plot of STRs frequency based on unit length.

Our search for STRs revealed 29,076 candidates located in 16,377 loci (23,759 transcripts) (Additional file 7: Table S5). From the discovered microsatellites, 12,625 STRs (~43%) are found in genes that code for proteins based on similarity searches with tilapia cDNA and 7,804 had a predicted ORF > 60 aa. The latter were used to study the STR distribution in the 5′UTR, 3′UTR and coding regions (Figure 5). The unit-length distribution of the discovered STRs is shown in Figure 6. Finally, STRs were found in 18 genes differentially expressed in brain and 3,713 in gonads.

## Discussion

The analyses presented explore the transcriptomic landscape of female and male gonad and brain tissues providing the first assessment of the molecular toolkit underlying the reproductive biology of a rudimentary hermaphrodite fish. We investigated the expression differences observed between sexes and tracked the expression of known targets of sex determination and differentiation. Finally, we provided a catalogue of SNPs and STRs that will assist following genetic research of the economically important sharpsnout seabream.

### The gonad and brain transcriptome of sharpsnout seabream

Gonad and brain expression profiling was conducted on males and females, with four individuals/biological replicates in each of the two groups. The reason we chose relatively immature individuals was two-fold. First, we sought to capture the transcriptomic differences between the two sexes in a stage where the two gonad types are clearly formed and sex is easily identifiable in this rudimentary hermaphrodite species. Second, we aimed at genes that contribute to sex differentiation, but upstream players that might play a role in sex determination as well. The stage selection was appropriate for characterizing the sex-related transcriptomic profiles of sharpsnout seabream, since we identified the great majority of previously reported sex differentiation and determination genes of various taxa in our assembled sequences.The general pattern observed in the expression analyses revealed a homogeneous global expression among brain samples for both sexes (Figure 2). On the contrary, gonads diverge greatly among individuals. Although, the two sexes clearly form two groups, there is great within-group variation. This might reflect the actual developmental stage of each individual. All individuals used in the study are of the same stage, but we cannot rule out the possibility that within the observed developmental stages different biological and molecular processes take place through a series of finer phases exhibiting alternative expression profiles.

### Male *vs*. female gonads expression patterns

The most striking aspect in the expression profiles of male and female gonads is the magnitude of the discovered differentially expressed genes (~20% of the total 71,387 loci expressed in gonads). A similar pattern is obtained from the PCA where the distances between the two gonad types are comparable to that between any of the two gonad types and the brain (Figure 2). This probably reflects their functional divergence. It is noteworthy that many more genes are over-expressed in male rather than female gonads (~60% of the gonad DE genes). This male-bias has been observed in other fish species gonad comparisons (zebrafish: [25, 26]; tilapia: [30]) or in whole organism male–female comparison (platyfish: [27]). However, given the lack of knowledge on the genetic and/or environmental mechanisms of sex determination and differentiation in sharpsnout seabream (or any other sparid), we cannot infer whether this is linked to the genetic architecture of the species or reflects other biological phenomena. The presence of genes expressed in the gonads of only one of the two sexes may be linked to the genetic architecture of sharpsnout seabream. The syntenic relationship of several of those genes observed in other species suggests a possible syntenic relationship in sharpsnout seabream and a common regulatory mechanism driven by the physical proximity on the genome. This can be tested in the future, e.g. with the construction of a genetic linkage map.

Multiple previous studies on vertebrates provide us with numerous candidate genes with possible involvement in sex determination and differentiation. A detailed search for those genes in the assembled transcriptome of sharpsnout seabream revealed that almost all are present, and some are differentially expressed in the gonads (Table 3). To start with the male phenotype, *Dmrt1* is the gene with the most prominent role in sex determination and spermatogenesis across vertebrates [74, 75]. In teleosts, it is highly linked with gonad development and maintenance (see [76] for a review). In Sparidae, *Dmrt1* has been implicated in the fate of the ovotestis in the protandrous black porgy *Acanthopagrus schlegelii*, and eliminating it results in a change from male to female [35, 77]. In our data, *Dmrt1* is up-regulated in male gonads implying a strong role in the development and maintenance of the male phenotype in the hermaphrodite sharpsnout seabream. *Amh*, and its receptor *Amhr2*, are both main candidates for triggering and maintaining the male phenotype. In our data, *Amh* is over-expressed in male gonads suggesting an important role in sharpsnout seabream. On the contrary, *Amhr2* exhibits similar expression levels in both gonad types. Both *Amh* and *Amhr2* have been linked to gonadal development in the protandrous black porgy [78]. Focused research on sharpsnout seabream and other Sparidae will reveal their specific role. *Amh* is tightly linked to *Sox9* and *Sf1* [79]. The male factor known to act on Sertoli cells, *Sox9*, was not differentially expressed in the sharpsnout seabream gonads. Other studies have showed that it is up-regulated in male Siberian sturgeons with undifferentiating gonads - in contrast to mature ones - [80], and it is over-expressed in the male immature gonads of sablefish [81]. Thus, *Sox9* might play a role in gonadal development at stages earlier than those studied here (e.g. see [82]). In contrast to *Sox9*, *Sf1* has significantly higher expression in male gonads. This factor might be tightly linked to the observed up-regulation of *Amh*. Male phenotype is reinforced by the high expression of androgen receptor, while *Dax1*- another gene involved in testis development- is similarly expressed between male and female gonads. The same pattern has been observed in tilapia [83], seabass [84] and medaka [85] where *Dax1* showed no expression difference suggesting a role in both gonad types. Interestingly, *Dhh*, one of the genes involved in testis differentiation and development in humans and mice [86, 87], is linked with the function of female rather than male gonads in sharpsnout seabream. However, in mammals it is expressed in both testes and ovaries [88–90]. Finally, multiple other genes strongly related with vertebrate male gonads are found within the sharpsnout seabream transcriptome (Additional file 3: Table S1).

Concerning the genes involved in female gonads, we observed significant over-expression in two key players of ovarian development, the ovarian aromatase (*Cyp19a1a*) and *β*-catenin *(Ctnnb). Cyp19a1* is a central component of ovarian steroidogenesis (converts androgens to estrogens). It is a conserved protein with a strong role in the ovarian development of vertebrates (see [91]). In fish, it is important not only for gonochoristic [92–95], but also hermaphrodite fish, as shown in [38] and confirmed in our data. The second activated key player, *Ctnnb*, is a member of the *Wnt4*/*β-catenin* pathway. However, *Wnt4* is not over-expressed in sharpsnout seabream female gonads. This pathway is well-studied in mammals (e.g. [96, 97]), but not much is known for teleosts; the few studies on teleosts show that *Wnt4* does not follow the pattern observed in mammals [23, 40, 98]. Finally, two genes that are known markers of ovarian development in mammals tightly linked to *Wnt4*/*β-catenin* pathway, R-spondin *(Rspo-1)* and Follistatin *(Fst)*, were not differentially expressed in sharpsnout seabream gonads. *Rspo-1* is involved in the ovarian differentiation in medaka in early stages of development; in later stages, its expression balances between male and female gonads [99]. *Fst* has been studied in detail in medaka [23] showing a lack of great differences among gonad types. Given the significant up-regulation of *Ctnnb*, the *Wnt4*/*β-catenin* pathway is involved in female gonads function and its significance and role should be deeper studied. Our search for *Foxl2*, another marker of ovarian development across vertebrates [100], showed that it is expressed similarly in both male and female gonads. This was also the case for the gonads of a protogynous hermaphrodite wrasse, *Halichoeres trimaculatus* [101], but not for several other gonochoristic fish [e.g. medaka [102, 103], tilapia [104], Southern catfish [105], catfish [106], Rare minnow [107], etc.], which suggests that *Foxl2* might play distinct roles in the gonads of hermaphrodite fishes. Finally, estrogen receptors did not exhibit any differentiation between the two sexes.

Genes known to have a role in sex determination and differentiation in vertebrates are active in the hermaphrodite sharpsnout seabream, as shown by the expression patterns in the current study. However, it is still open whether the molecular pathways are conserved compared to other teleosts. The current knowledge and comparisons among teleosts show that both upstream and downstream genes alter their position and function in space and time [23, 95]. Apart from the known candidates, we observed thousands other genes in our data. Those reflect the complex biological processes taking place in the gonads (e.g. cell proliferation, metabolic processes, regulation of transcription, etc.).

### Male *vs*. female brain expression patterns

Brain has a pronounced sexual dimorphism in function in mammals. However, even in model organisms like human, detailed information is only recently being gathered e.g. [108]. In fish brain, sexual dimorphism is less pronounced in gonochorists compared to mammals, and even less in hermaphrodites [109]. Further, the teleost brain is characterized by remarkable sexual plasticity [110]. Our results showed that in sharpsnout seabream, male and female brains have almost identical expression patterns with few exceptions. The observed divergence was smaller than reported in other fishes (e.g. zebrafish: [25]; rainbow trout: [111]; medaka: [109]). This may be due to reduced sex-specificity in rudimentary hermaphrodites brain.

The discovered genes provide a baseline for understanding the brain sexual divergence in sharpsnout seabream (Figure 4). Like in gonads, more genes are over-expressed in male rather than female brains. These genes are involved in development, metabolism, regulation of transcription, vision, etc. Some are even involved in RNA-dependent DNA replication -probably linked to transposable elements. Notably, none of the known sex determining genes is differentially activated in the brain. The genes over-expressed in male brains include several factors linked to sex in other taxa, such as *Tcf12* (involved in estrogen/antiestrogen response in the teleost Fathead minnow [112]), *Cbln1* (over-expressed in mouse testis compared to ovaries, as part of the male developmental pathway [113–116]), *Rs1* (X-linked gene in human associated to a common macular degeneration in males [117]), *Ca12* (involved in the function of uterus in mice [118]), *Hyou1* (involved in gonadogenesis in mice [119]) and *Aqp1* (possible involvement in the water homeostasis of the male reproductive system [120]). From the genes over-expressed in females, *Hamp* is regulated by estrogens in mice [113] regulating brain iron metabolism [121]. Other genes that might play significant but still unknown roles in the development of female brain are *Alx3*, *NeoVTX subunit alpha*, *Thap9* and *Itga2*. All those are candidates for regulating and maintaining the sex-specific brain phenotypes and require deeper investigation.

In Sparidae, brain expression has been studied in black porgy; the expression of three *Gnrh* genes in different developmental stages of ovaries and testis has been characterized and they were found linked to sexual development [38]. However, in sharpsnout seabream all three genes (*Gnrh-I-III*) have only basal or no expression in both male and female brains. Further, in the gonads we found significant divergence in the expression of aromatase *α*, which had no expression in either male or female brains. On the contrary, we found strong expression of aromatase *β* in the brain and low expression in the gonads of both sexes. In both tissues, no significant expression difference was observed between the sexes for aromatase *β*. Those are the expected expression patterns of the two aromatases as observed in other teleosts as well (summarized in [48]). Targeted studies and comparative data from other species will elucidate the role each of those genes play and the mechanisms regulating their expression (e.g. environmental, social, genetic, etc.).

### Genetic marker discovery through RNA-sequencing

The possibility to reconstruct the expressed genic sequences at a global scale through RNA-Seq, gives access to a plethora of genetic markers widely distributed in the genome. We identified a considerable dataset of STR and SNP markers that can be used as raw material in future population genetics, broodstock management, QTL mapping and Marker Assisted Selection (MAS) in sharpsnout seabream and other phylogenetically close sparid species. Discovering SNP markers can be a relatively easy task in a dataset like the one produced here, as pooling individuals allows identifying polymorphic sites robustly (although this can be proven only through further targeted experiments). However, further filtering of the SNPs for individual genotyping (see Methods) reduced dramatically the available SNPs. Thus, finding SNP markers that pass certain quality filters for all sequenced individuals is not trivial. To increase the number of informative markers, either deeper sequencing of each individual or use of different genotyping methods would be more appropriate (e.g. RNA-Seq with normalized libraries, Genotyping by Sequencing, SNP-chips scanning, etc.). Apart from SNPs, RNA-Seq leads to the discovery of thousands new STR markers in the transcribed regions of the genome, offering the possibility to select appropriate markers for future analyses.

## Conclusions

In this study, we conducted a comprehensive search of the genes expressed in the male and female gonad and brain tissues of sharpsnout seabream. We used the information extracted from sequencing the RNA of the two tissue types i) to obtain a global view of the sex-specific expression patterns in a rudimentary hermaphrodite teleost and ii) to gather transcriptomic information that will make future comparative analyses feasible. The picture we obtained refers to the particular developmental stage, and averages among all sub-tissues within brain or gonads. In our results, we found the same genes that are responsible for sex differentiation in numerous other species. Some of them might play a role in the sex determination procedure that takes place early in the development. The whole-transcriptome approach employed lays the foundation for future studies to identify genes responsible not only for the sex determination, but also the gonad development and maintenance in sharpsnout seabream. Similar analyses on the tissue parts and of different developmental stages will provide the dynamic view necessary for a complete understanding of sex development.

Eventually, comparison of the expression profiles in sharpsnout seabream with closely related species that display alternative reproductive modes (e.g. the protandrous *Sparus aurata* and other protogynous species), will offer impressive insights into the particular genetic toolkits deployed in each mechanism.

## Availability of supporting data

Raw reads are deposited in N.C.B.I. sequence read archive under the BioProject ID PRJNA241484.

## Electronic supplementary material

Additional file 1: Figure S1: Female gonad tissue RNA profile. Total RNA profile of a female gonad as retrieved from an agarose gel and the Agilent Bioanalyzer. (PDF 565 KB)

Additional file 2: Figure S2: MA plot for gonad and brain samples. Axes represent log_2_ fold change versus the mean normalized base counts; significantly differentially expressed loci are shown in red. Genes exceeding the axes range are not shown. (PDF 11 MB)

Additional file 3: Table S1: The list of differentially expressed genes in gonad and brain tissues (FDR < 0.05). (XLSX 2 MB)

Additional file 4: Table S2: GO terms over-represented in female or male up-regulated genes in sharpsnout seabream gonads. Fisher’s exact test (adjusted p-value < 005) on the GO terms of male versus female gonads over-expressed loci. (XLSX 40 KB)

Additional file 5: Table S3: Genes associated with sex-related GO terms. (XLSX 54 KB)

Additional file 6: Table S4: Comparative analysis of genes expressed only in the gonads of one sex in sharpsnout seabream. (XLSX 55 KB)

Additional file 7: Table S5: SNP and STR markers discovered. (XLSX 10 MB)

Additional file 8: Table S6: Individual genotyping for the high quality selected SNP loci. (XLSX 127 KB)

Additional file 9: Figure S3: PCA conducted on SNP genotypes. The genotypes of each individual from the restricted set of 1009 SNPs are analyzed in a PCA. (PDF 87 KB)

Additional file 10: Table S7: Kinship coefficients of the studied individuals. (XLSX 51 KB)
