# Supplementary figures and images for: The sex-specific transcriptome of the hermaphrodite sparid sharpsnout seabream (Diplodus puntazzo)
Source: BMC Genomics. 2014 Aug 6;15:655. doi: 10.1186/1471-2164-15-655 (PMC4133083; doi:10.1186/1471-2164-15-655)

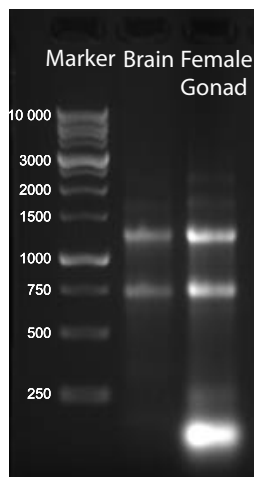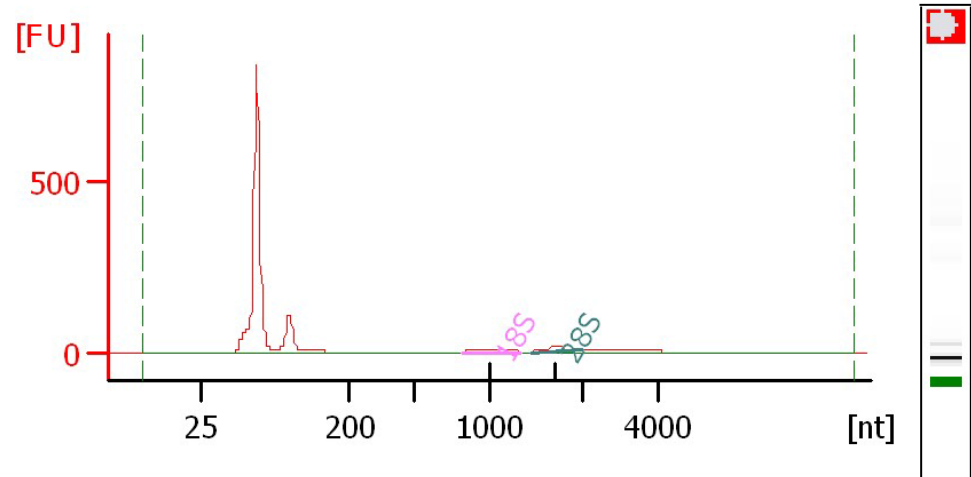

Supplement: Supplementary file 1 — Additional file 1: Figure S1: Female gonad tissue RNA profile. Total RNA profile of a female gonad as retrieved from an agarose gel and the Agilent Bioanalyzer. (PDF 565 KB) [file 12864_2014_6346_MOESM1_ESM.pdf]

**A. Gonads MA plot**

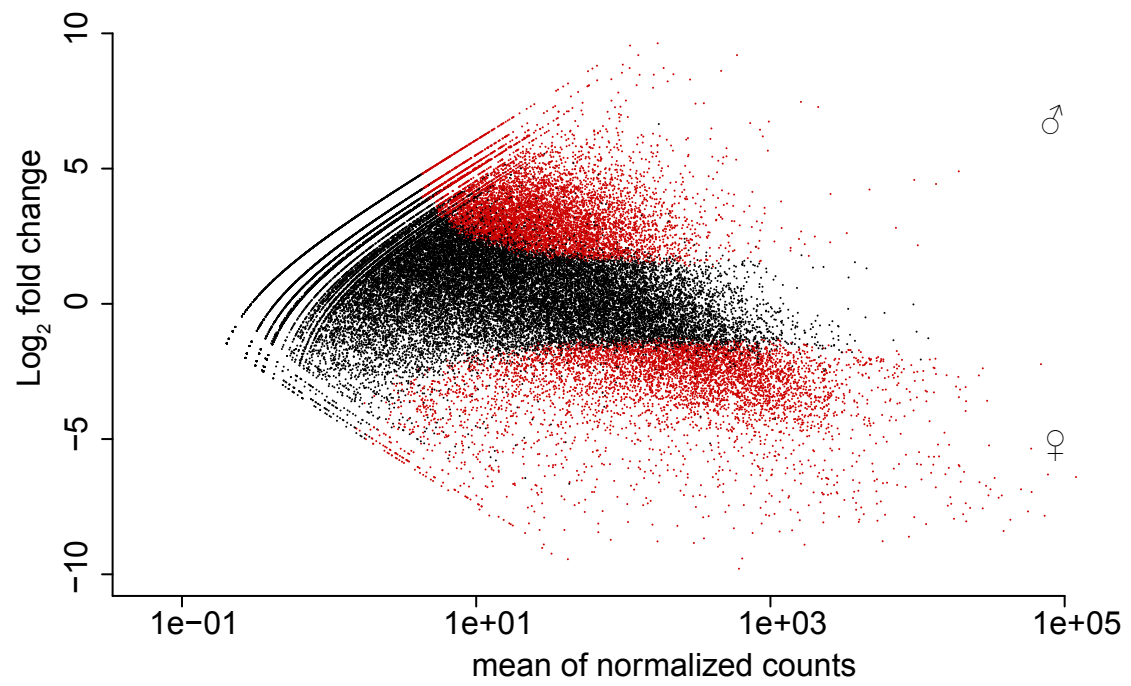

**B. Brains MA plot**

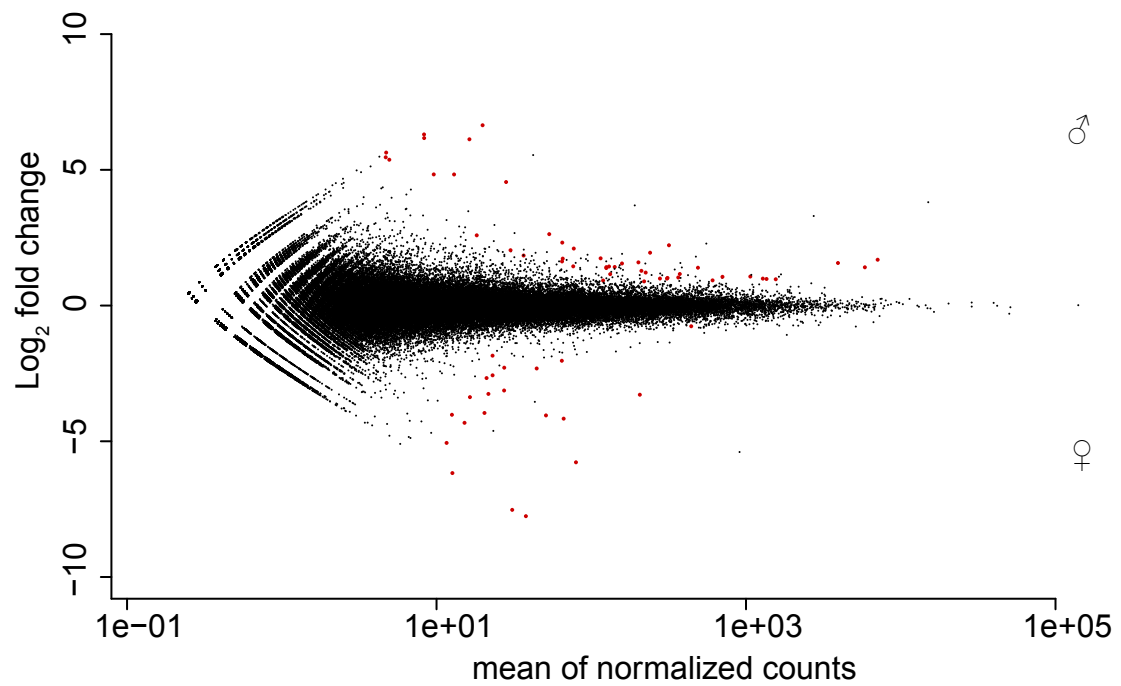

Supplement: Supplementary file 2 — Additional file 2: Figure S2: MA plot for gonad and brain samples. Axes represent log2 fold change versus the mean normalized base counts; significantly differentially expressed loci are shown in red. Genes exceeding the axes range are not shown. (PDF 11 MB) [file 12864_2014_6346_MOESM2_ESM.pdf]

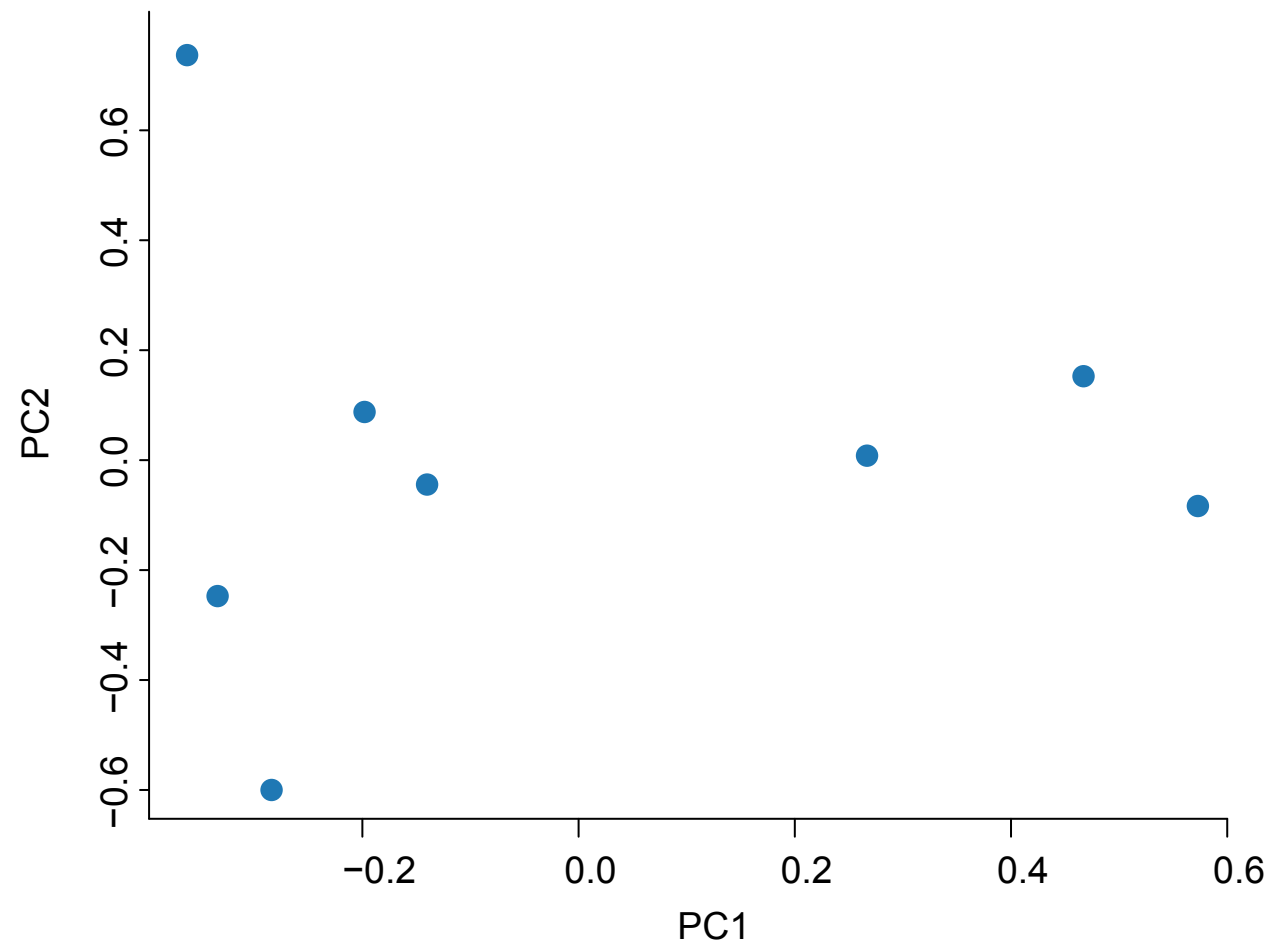

Supplement: Supplementary file 9 — Additional file 9: Figure S3: PCA conducted on SNP genotypes. The genotypes of each individual from the restricted set of 1009 SNPs are analyzed in a PCA. (PDF 87 KB) [file 12864_2014_6346_MOESM9_ESM.pdf]
